# GENOMOS Study Finds Weak Links between *COL1A1* Polymorphism, BMD, and Fracture Risk

**DOI:** 10.1371/journal.pmed.0030152

**Published:** 2006-02-21

One out of every two women and one in eight men over 50 will have an osteoporosis-related fracture in their lifetime. Osteoporosis is characterized by low bone mass and structural deterioration of bone tissue, and often progresses without overt symptoms or pain until a bone breaks. Fractures occur typically in the hip, spine, and wrist. Currently, there is no accurate measure of overall bone strength. Bone mineral density (BMD) is frequently used as a proxy measure, but it can explain only a modest proportion of fracture risk.

Bone resorption and bone formation take place throughout life. Formation outpaces resorption until peak bone mass (maximum bone density and strength) is reached around age 30. From then on, bone resorption slowly begins to exceed bone formation, and the balance is further shifted toward resorption in women after menopause. Osteoporosis develops when bone resorption occurs too quickly or replacement too slowly—which happens in most individuals at a certain age and often earlier in individuals who did not reach optimal bone mass during their bone building years.

Both bone formation and resorption are under the control of genetic and environmental factors. Osteoporosis is a “complex disease,” with variations in a number of different genes and in several environmental factors (such as calcium intake or alcohol consumption) thought to affect an individual's risk. A number of candidate genes have been identified, some of them through studies of rare genetic diseases affecting bone health, others through animal studies. They include genes for calciotropic hormones and their receptors, as well as bone matrix proteins.

One of them, *COL1A1*, encodes collagen 1 alpha 1, a major component of bone and cartilage. Mutations in its coding region cause osteogenesis imperfecta, a rare developmental bone disorder characterized by brittle bones, frequent fractures, and short stature. Apart from these rare mutations, *COL1A1* has a number of polymorphic sites outside the coding region, and scientists have examined associations between many of these alleles and osteoporosis. The one that has been studied most intensely is a single-nucleotide polymorphism within the promoter region at a binding site for the Sp1 transcription factor. The more common allele has a guanine nucleotide (G) at the variable position; the rarer one, a thymine (T). In vitro studies suggest that the T allele is associated with less transcript and protein produced.

Several previous studies had examined a possible association between the T allele and low bone mineral density and fractures, and a number of them had found such a link, as had three separate meta-analyses. As a consequence, some researchers have suggested that genetic testing at the population level for this polymorphism would be beneficial. Individuals who carry the T allele could be advised to get enough calcium and do weight-bearing exercises, ideally already during the bone acquisition phase in adolescence. Others have warned that the evidence that links the T allele to a higher risk for osteoporosis is not strong enough to support such action. They have pointed out some of the notorious problems with association studies in general and retrospective meta-analyses based on published studies.

The Genetic Markers for Osteoporosis (GENOMOS) project is a European Union–funded European collaborative research initiative between universities in the Netherlands, United Kingdom, Italy, Spain, Greece, Poland, and Denmark. The project, which began in 2003, currently involves around 24,000 individuals and seeks to identify genetic risk factors for osteoporosis by prospective meta-analysis. Participants have been recruited from a total of 18 European countries (most of them reside in the UK, the Netherlands, Spain, Italy, Denmark, and Poland). Data on previous and new fractures are collected, together with bone densitometry measurements, information on risk factors, and DNA analysis from blood samples.

The GENOMOS investigators, led by John Ioannidis, report now on their examination of an association between the Sp1 polymorphism in *COL1A1*, BMD, and fracture risk. Based on data from over 20,000 participants, they found a modest association between homozygosity for the T allele and lower bone mineral density at the femoral neck and the lumbar spine. The researchers also found a weak association between the T allele and vertebral fractures in women. However, T allele carriers did not have an overall increased risk of fractures.

The effects seen in this large study—GENOMOS participants reported more than five times the number of fractures than participants in all previous studies combined—were more moderate than those reported in most of the earlier studies. The Sp1 polymorphism in *COL1A1* explained only a small part of the differences in BMD and fracture risk among the GENOMOS participants. There was no association between the T allele and BMD in heterozygous carriers (and only approximately 4% of the participants were homozygous for the T allele). Regarding the fracture association, the researchers estimate that the presence of the T allele would explain at most 10% of the risk of vertebral fractures for women.

The authors conclude that “large-scale studies are needed to quantify the true effect size of genetic polymorphisms that have been implicated in the pathogenesis of complex genetic disorders.” Their findings also argue against widespread genetic testing for this particular polymorphism alone. Researchers need to look at other genes (and possibly other variants in the *COL1A1* gene) and validate any findings in large studies like this one before they can predict a substantial fraction of a random individual's genetic risk for osteoporosis.

## 

**Figure:**
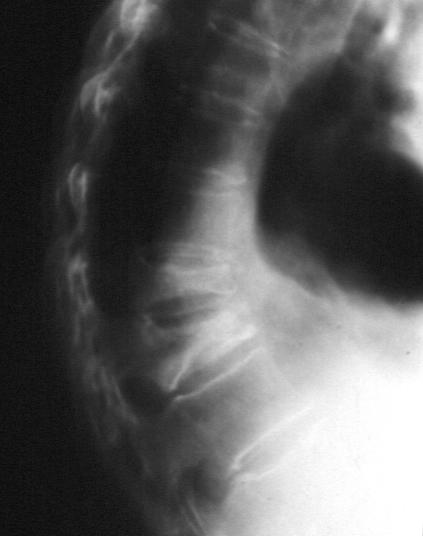
Vertebral compression fractures in a patient with osteoporosis

